# Amelioration of olfactory dysfunction in a mouse model of Parkinson’s disease via enhancing GABAergic signaling

**DOI:** 10.1186/s13578-023-01049-9

**Published:** 2023-06-03

**Authors:** Xing-Yang Liu, Ke Wang, Xian-Hua Deng, Yi-Hua Wei, Rui Guo, Sui-Feng Liu, Yi-Fan Zhu, Jia-Jun Zhong, Jing-Yuan Zheng, Meng-Dan Wang, Qiu-Hong Ye, Jian-Quan He, Kai-Hang Guo, Jun-Rong Zhu, Shu-Qiong Huang, Ze-Xu Chen, Chong-Shan Lv, Lei Wen

**Affiliations:** 1 State Key Laboratory of Cellular Stress Biology, Fujian Provincial Key Laboratory of Neurodegenerative Disease and Aging Research, Institute of Neuroscience, Xiang’an Hospital, School of Medicine, Xiamen University, Xiamen, Fujian 361102 China; 2 Xiamen Key Laboratory for TCM Dampness Disease, Neurology & Immunology Research, Department of Traditional Chinese Medicine, Longyan Hospital of Traditional Chinese Medicine, School of Medicine, Xiamen University, Longyan, Fujian 364000 China; 3 Zhongshan Hospital, School of Medicine, Xiamen University, Xiamen, Fujian 361100 China

**Keywords:** Parkinson’s disease, Olfactory dysfunction, GABAergic signaling, Excitation/inhibition imbalance

## Abstract

**Background:**

Olfactory dysfunction is among the earliest non-motor symptoms of Parkinson’s disease (PD). As the foremost pathological hallmark, α-synuclein initiates the pathology in the olfactory pathway at the early stage of PD, particularly in the olfactory epithelium (OE) and olfactory bulb (OB). However, the local neural microcircuit mechanisms underlying olfactory dysfunction between OE and OB in early PD remain unknown.

**Results:**

We observed that odor detection and discrimination were impaired in 6-month-old SNCA-A53T mice, while their motor ability remained unaffected. It was confirmed that α-synuclein increased and accumulated in OB but not in OE. Notably, the hyperactivity of mitral/tufted cells and the excitation/inhibition imbalance in OB were found in 6-month-old SNCA-A53T mice, which was attributed to the impaired GABAergic transmission and aberrant expression of GABA transporter 1 and vesicular GABA transporter in OB. We further showed that tiagabine, a potent and selective GABA reuptake inhibitor, could reverse the impaired olfactory function and GABAergic signaling in OB of SNCA-A53T mice.

**Conclusions:**

Taken together, our findings demonstrate potential synaptic mechanisms of local neural microcircuit underlying olfactory dysfunction at the early stage of PD. These results highlight the critical role of aberrant GABAergic signaling of OB in early diagnosis and provide a potential therapeutic strategy for early-stage PD.

**Supplementary Information:**

The online version contains supplementary material available at 10.1186/s13578-023-01049-9.

## Background

Parkinson’s disease (PD) is a neurological disorder characterized by the classical motor features of parkinsonism associated with Lewy bodies and loss of dopaminergic neurons in the substantia nigra pars compacta (SNc). However, the symptomatology of PD is now recognized as heterogeneous, with clinically significant non-motor features [1–3]. Growing evidence has identified olfactory dysfunction as one of the earliest non-motor symptoms seen in over 90% of PD patients, seriously compromising their quality of life. Furthermore, plenty of researches have revealed that olfactory dysfunction can predate motor symptoms by over 10 years and predict a phenotype with worse motor function and cognition [4–6]. Nevertheless, the underlying mechanisms of olfactory dysfunction in PD remain elusive.

In the olfactory system, odor information is initially captured and transduced into electrical signals by the olfactory sensory neurons (OSNs) residing in the olfactory epithelium (OE). Subsequently, these signals are transmitted along the axons of the OSNs to the olfactory bulb (OB), where they are decoded by mitral/tufted cells (M/Ts) and numerous interneurons before being projected to the higher olfactory cortex [7–9]. During Braak stages I and II of PD, α-synuclein has been detected in the OB and anterior olfactory nucleus [10]. Moreover, it has been reported that α-synuclein deposits and local neural network disharmony in OB occur earlier than those in SNc [11]. Notably, mutant α-synuclein (A53T or A30P) has a higher propensity to form aggregates than wild-type α-synuclein and can cause dominant familial PD [12–15]. A recent study demonstrated that overexpression of α-synuclein targeted in OB led to increased α-synuclein aggregates and olfactory impairments, which could underscore the critical role of OB in olfactory information processing [16]. Additionally, recent investigations have identified α-synuclein seeds in the OE of early-stage PD patients through the real-time quaking-induced conversion (RT-QuIC) assay [17–19]. However, the relationships between the initial site of α-synuclein deposits in OE or OB and the local neural microcircuit mechanisms underlying olfactory dysfunction in early PD remain poorly understood, significantly impacting the early diagnosis and treatment of PD.

To elucidate the precise neural microcircuitry mechanisms underlying olfactory dysfunction associated with mutant α-synuclein between OE and OB, we investigated the primary olfactory pathway using a mouse model that overexpressed the human A53T mutant α-synuclein. Our findings revealed that 6-month-old SNCA-A53T mice exhibited olfactory impairments despite normal motor function. We also found that α-synuclein increased and accumulated in OB, while no α-synuclein staining was detected in OE, accompanied by an unaffected OSN number in OE. Meanwhile, no detectable change in tyrosine hydroxylase (TH)-positive neurons was found in the SNc and striatum (STR) in 6-month-old SNCA-A53T mice. In addition, we discovered that, GABAergic transmission was impaired in OB, with hyperactivity of M/Ts and excitation/inhibition (E/I) imbalance in OB of SNCA-A53T mice. We further showed that, the aberrant expression of GABA transporter 1 (GAT1) and vesicular GABA transporter (VGAT) may contribute to the impaired GABAergic transmission in OB. Finally, we demonstrated that, tiagabine (TGB), a potent and selective GABA reuptake inhibitor, could attenuate the aberrant neural microcircuitry in OB and reverse the impaired olfactory function in SNCA-A53T mice. Our results provide evidence of the potential diagnosis and therapy of early PD.

## Results

### Impaired olfactory behaviors in 6-month-old SNCA-A53T mice

**Fig. 1:**
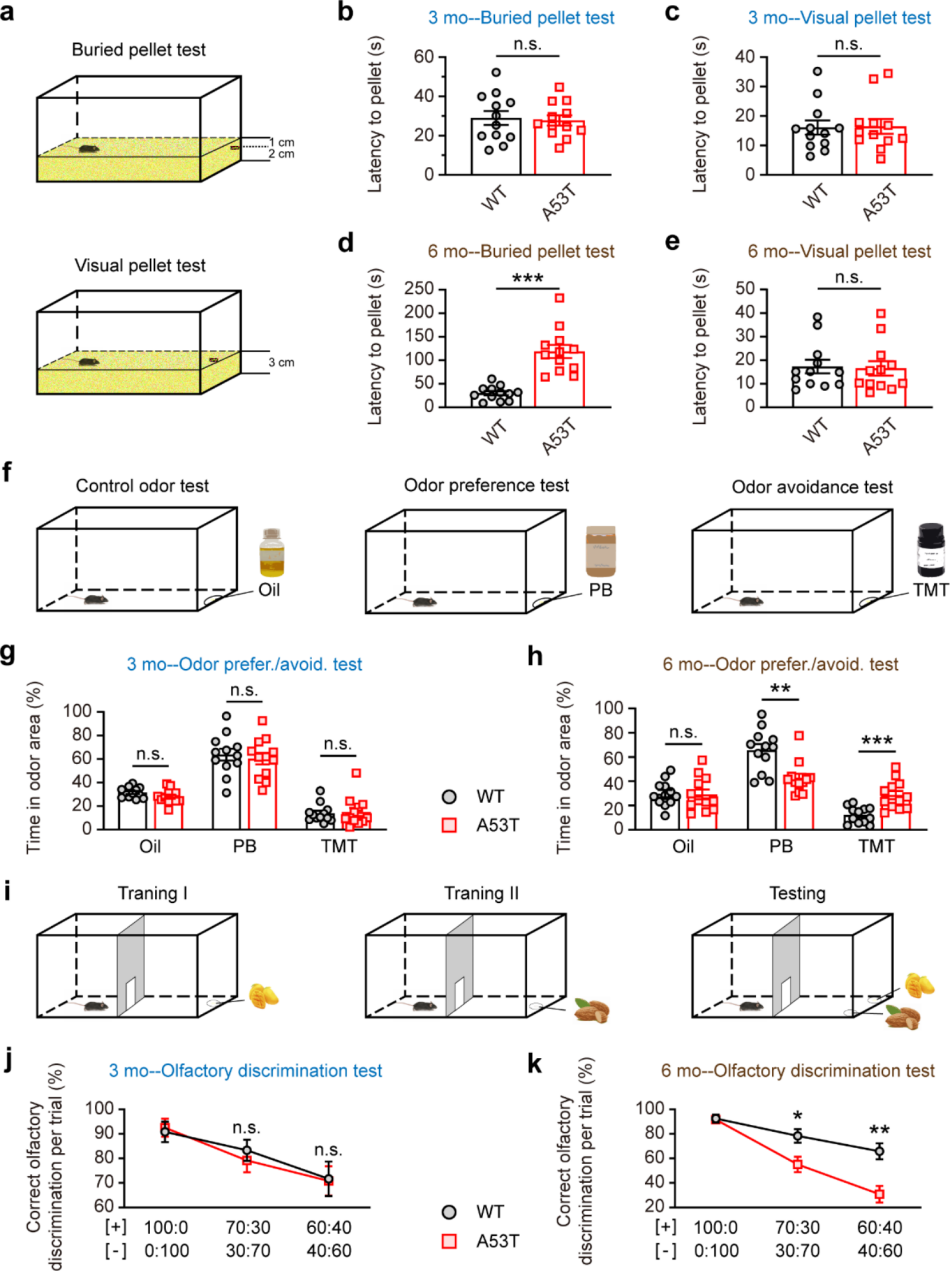
Impaired olfactory behaviors of 6-month-old SNCA-A53T mice. **a** Schematic diagram illustrating the buried and visual food tests. **b-e** Quantification of latency to find a food pellet buried (**b, d**) or on the surface (**c, e**) of bedding in 3-month-old and 6-month-old WT and A53T mice (n = 12 for each group). **f** Schematic diagram of olfactory preference/avoidance test. **g-h** Percentage of time spent on sniffing the odor area containing oil, PB, and TMT, in 3-month-old (**g**) and 6-month-old (**h**) WT and A53T mice (n = 12 for each group). **i** Schematic diagram showing the olfactory discrimination test. **j-k** Percentage of correct olfactory discrimination per trial session in 3-month-old (**j**) and 6-month-old (**k**) WT and A53T mice (n = 12 for each group). Data are presented as mean ± SEM. *p < 0.05; **p < 0.01; ***p < 0.001; n.s., not significant

Since olfactory impairments occur at the early stage of the neurodegenerative process of PD [4, 5], we investigated the development of olfactory dysfunction in SNCA-A53T mice. Firstly, we performed a buried food pellet test to measure olfactory sensitivity in 3-month-old and 6-month-old mice (Fig. 1a). We found that 6-month-old, rather than 3-month-old SNCA-A53T mice took more time than their age-matched wild-type (WT) mice to locate the buried food pellet (Fig. 1b, d), indicating possible impairment of odor detection in 6-month-old SNCA-A53T mice. To exclude the potential influence of locomotion, we carried out a visual food pellet test, which showed that these groups of mice spent similar time obtaining food pellets on the bedding (Fig. 1c, e), indicating that their motivation was unaffected. Next, we conducted an olfactory preference/avoidance test to assess the ability to recognize fond or aversive odors (Fig. 1f). Peanut butter (PB) and 2,4,5-trimethylthiazole (TMT) were used as the fond and aversive odors, respectively. The time each mouse spent sniffing the filter paper containing the odor solution was recorded. Each mouse was first tested with filter paper containing mineral oil to eliminate the influence of the filter paper and solvent. The results showed no significant differences among the groups. Additionally, we observed that 6-month-old SNCA-A53T mice spent less time investigating the filter paper containing PB than their age-matched WT mice, and they took more time to sniff the filter paper containing TMT than their age-matched WT mice. However, no significant difference in the PB and TMT solution tests was observed between 3-month-old SNCA-A53T and WT mice (Fig. 1g, h). These results indicate a deficit in the innate response to fond or aversive odor in 6-month-old SNCA-A53T mice. Furthermore, we performed an olfactory discrimination test to assess the ability to distinguish between different fractions of mango and almond smells (Fig. 1i). Compared to age-matched WT mice, the percentage of correct olfactory discrimination responses per trial in different fractions of smell mixtures was reduced in 6-month-old but not in 3-month-old SNCA-A53T mice (Fig. 1j, k), indicating olfactory discrimination impairment in 6-month-old SNCA-A53T mice.

In addition, we evaluated the motor ability of SNCA-A53T and WT mice at 3 and 6 months of age via a rotarod test (Additional file 1: Fig. S1a). The latency period until the mice fell to the ground was assessed in the trial. There were no significant differences in rotarod performance between SNCA-A53T and age-matched WT mice (Additional file 1: Fig. S1b, c). Additionally, we performed a pole test (Additional file 1: Fig. S1d) and found that the durations of descending the pole were similar between SNCA-A53T and age-matched WT mice (Additional file 1: Fig. S1e, f). Moreover, we conducted a hanging wire test (Additional file 1: Fig. S1g), and the variable trends in the results were consistent with those of the rotarod and pole tests (Additional file 1: Fig. S1h, i). Taken together, these results showed that SNCA-A53T mice did not exhibit motor dysfunction at 3 and 6 months old. As we confirmed that olfactory dysfunction in SNCA-A53T mice started to occur at 6 months of age, we subsequently focused on 6-month-old mice to explore the underlying mechanisms of olfactory dysfunction unless otherwise noted.

### Unchanged α-synuclein level and cytoarchitecture in the OE of SNCA-A53T mice

**Fig. 2:**
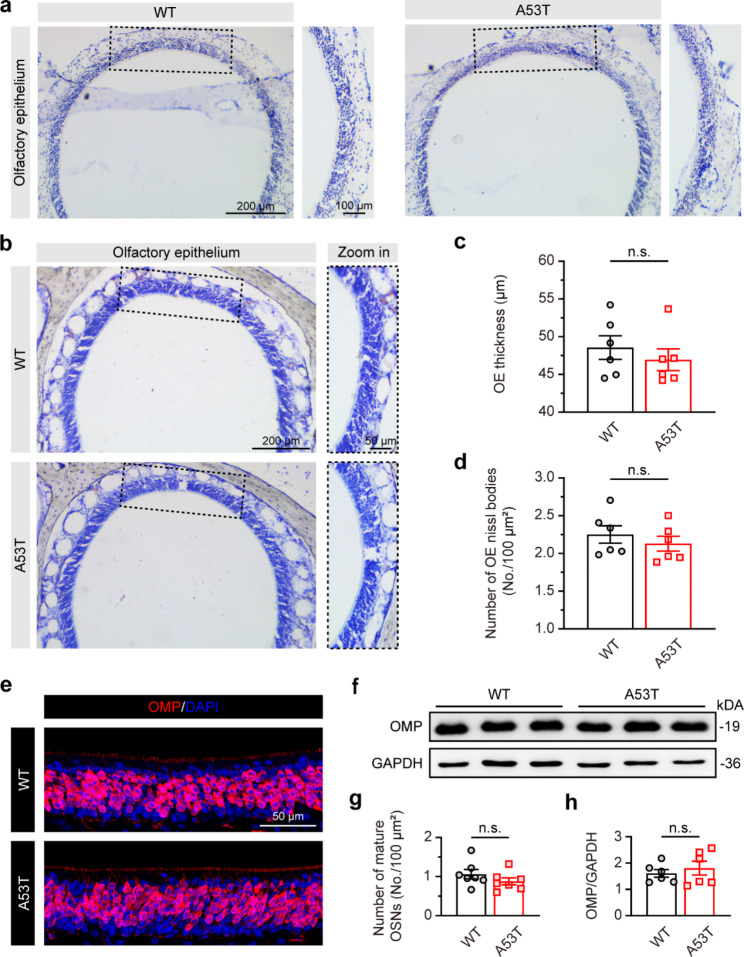
Unchanged α-synuclein expression and cytoarchitecture in OE of 6-month-old SNCA-A53T mice. **a** Representative images showing α-synuclein immunostaining of OE. **b** Representative images showing Nissl staining of OE. **c-d** Quantification of the thickness (**c**) and number of Nissl bodies (**d**) of OE in WT and A53T mice (n = 3 for each group). **e-f** Representative images showing OMP immunostaining (**e**) and immunoblotting (**f**) in OE. **g-h** Quantification of the number of mature OSNs (OMP-positive neurons) (**g**, n = 3 for each group) and the protein levels of OMP (**h**, n = 6 for each group) in OE of WT and A53T mice. Data are presented as mean ± SEM. n.s., not significant

Although previous studies have demonstrated that α-synuclein may seed in the OE [17–19], the gatekeeper of the primary olfactory pathway, it remains unclear whether α-synuclein is first triggered in the OE when olfactory dysfunction occurs. Therefore, we first performed α-synuclein immunostaining in the OE. Surprisingly, we found a similar level of α-synuclein in OE between SNCA-A53T and WT mice (Fig. 2a). We then performed Nissl staining in OE (Fig. 2b) and found no significant differences in the thickness and number of Nissl bodies (indicated as neurons) between SNCA-A53T and WT mice (Fig. 2c, d). We further performed olfactory marker protein (OMP) immunofluorescent staining and immunoblotting in OE to assess the mature OSNs (Fig. 2e, f). The results showed that there were no significant differences in OMP-positive neurons or OMP levels between SNCA-A53T and WT mice (Fig. 2g, h). These findings demonstrate that the cytoarchitecture of OE remains uninjured whereas olfactory dysfunction occurs in SNCA-A53T mice.

### α-Synuclein accumulation in the OB of SNCA-A53T mice

**Fig. 3:**
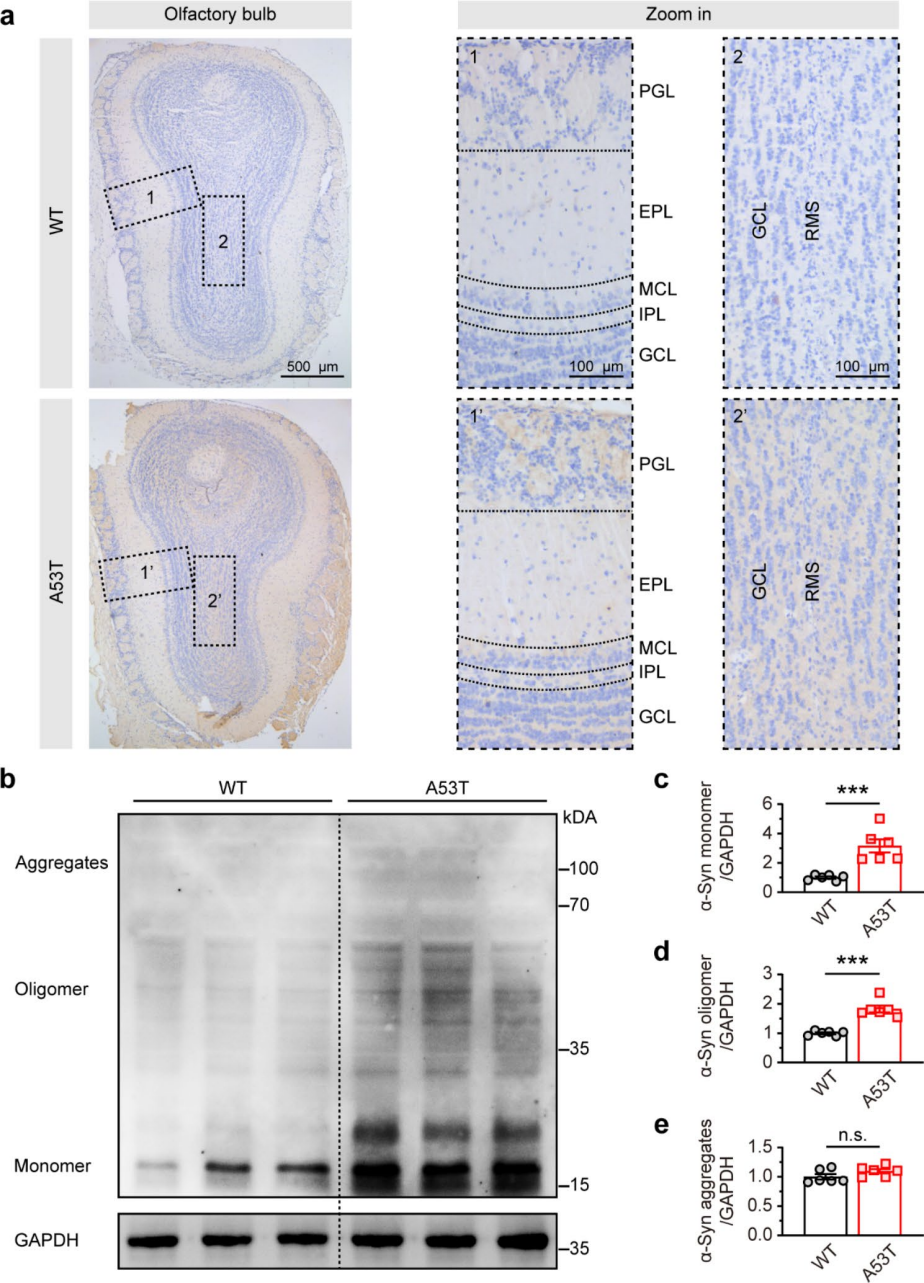
Increased and accumulated α-synuclein in OB of 6-month-old SNCA-A53T mice. **a** Representative images showing α-synuclein immunostaining in different layers of OB in WT and A53T mice. **b** Representative image showing α-synuclein immunoblotting of OB in WT and A53T mice. **c-e** Quantitative analysis of the expression of α-synuclein monomer (**c**), oligomer (**d**), and aggregates (**e**) in OB of WT and A53T mice (n = 6 for each group). Data are presented as mean ± SEM. ***p < 0.001; n.s., not significant

Based on the dual-hit hypothesis of PD, α-synuclein is triggered in the OB and subsequently spreads to other areas [10]. Recent evidence has demonstrated that OB is susceptible to α-synuclein and that α-synuclein aggregates restricted to the OB can induce olfactory impairment, strongly suggesting that α-synuclein in the OB is closely correlated with olfactory dysfunction [16, 20]. Therefore, we further investigated the spatial distribution characteristics of α-synuclein in the OB by immunostaining in SNCA-A53T and WT mice. Our results showed that α-synuclein was prominent in the OB of SNCA-A53T mice, whereas WT mice exhibited only light staining. α-Synuclein deposits were observed in each layer of the OB, but the periglomerular layer (PGL) was more evident than other layers (Fig. 3a). Additionally, western blotting analysis demonstrated that, α-synuclein monomers and oligomers, but not aggregates, were significantly increased in the OB (Fig. 3b-e). Thus, our findings suggest that α-synuclein accumulation occurs earlier in the OB than in the OE in SNCA-A53T mice.

Meanwhile, given that dyskinesia in PD is closely correlated with the number of TH-positive neurons in SNc and STR, we further investigated whether the number of TH-positive neurons changed in the SNc and STR of 6-month-old SNCA-A53T mice. Histological analysis revealed a similar number of TH-positive neurons in the SNc and STR between SNCA-A53T and WT mice (Additional file 1: Fig. S2a-c), which suggested that the typical pathological changes associated with the loss of dopamine neurons in the SNc and STR remained undetectable in 6-month-old SNCA-A53T mice, in accordance with the above results in motor behavioral experiments.

### Excitation/inhibition imbalance in the OB of SNCA-A53T mice

**Fig. 4:**
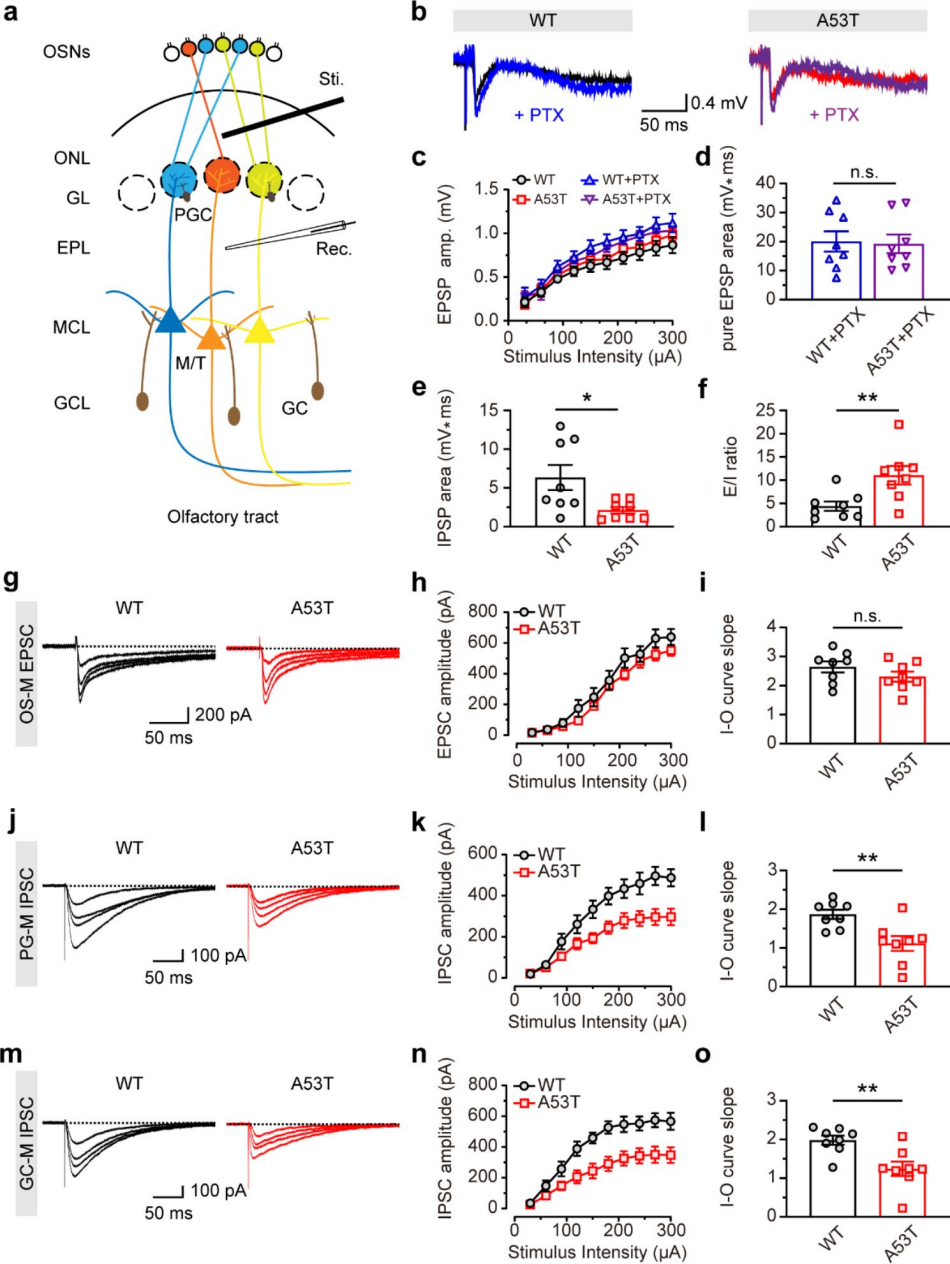
The excitation/inhibition imbalance in OB of 6-month-old SNCA-A53T mice. **a** Schematic diagram illustrating the fEPSP recording in OB. **b** Representative images showing mixed (indicated as black and red traces) and pure (indicated as blue and purple traces) fEPSP in OB of WT and A53T mice. **c** Input-output curves of mixed and pure fEPSP in OB of WT and A53T mice (n = 4 for each group). **d-f** Quantitative analysis of averaged pure fEPSP area (**d**), averaged area of fIPSP (**e**) and the ratio of E/I (**f**) in OB of WT and A53T mice under the last three stimuli (n = 4 for each group). **g-h** Representative images (**g**) and input-output curves (**h**) of OE-M EPSCs of M/Ts in OB of WT and A53T mice (n = 4 for each group). **i** Quantitative analysis of the slope of (**h**) in WT and A53T mice (n = 4 for each group). **j-k** Representative images (**j**) and input-output curves (**k**) of PG-M IPSCs of M/Ts in OB of WT and A53T mice (n = 4 for each group). **l** Quantitative analysis of the slope of (**k**) in WT and A53T mice (n = 4 for each group). **m-n** Representative images (**m**) and input-output curves (**n**) of GC-M IPSCs of M/Ts in OB of WT and A53T mice (n = 4 for each group). **o** Quantitative analysis of the slope of (**n**) in WT and A53T mice (n = 4 for each group). Data are presented as mean ± SEM. *p < 0.05; **p < 0.01; n.s., not significant

Growing evidence suggests that OB plays a critical role in maintaining normal olfactory function, and the E/I balance in the OB can affect olfactory processing [21]. We further conducted electrophysiological recordings in the OB slices to investigate the neural microcircuitry characteristics of the OB in SNCA-A53T and WT mice. The M/Ts of the OB are projection neurons that receive glutamatergic upstream innervation from OSNs of the OE and GABAergic feedback inhibition from interneurons of the OB. To assess the function of OB neural microcircuitry, the olfactory nerve layer (ONL) containing axonal terminals of OSNs was stimulated, and the field excitatory postsynaptic potentials (fEPSPs) were recorded in the external plexiform layer (EPL), which contained dendritic terminals of M/Ts in OB slices (Fig. 4a, b and Additional file 1: Fig. S3). In the presence or absence of the GABA receptor antagonist, picrotoxin, fEPSPs were divided into pure and mixed fEPSPs (Fig. 4c). The results showed that the area of pure fEPSPs was similar in OB slices of SNCA-A53T and WT mice (Fig. 4d), indicating an unchanged OSN-induced excitatory response of M/Ts. Since the picrotoxin-sensitive components represent GABAergic feedback inhibition from GABAergic interneurons of OB, the field inhibitory postsynaptic potentials (fIPSPs) were evaluated by subtracting the area of mixed fEPSPs from pure fEPSPs. Interestingly, we found that, compared with WT mice, the area of fIPSPs significantly decreased in SNCA-A53T mice (Fig. 4e), suggesting weaker inhibitory responses of M/Ts in SNCA-A53T mice. Moreover, an increased E/I ratio indicated an E/I imbalance in OB of SNCA-A53T mice (Fig. 4f).

We further analyzed the function of specific synapses, including excitatory OS-M, inhibitory PG-M, and inhibitory GC-M synapses. To investigate the function of the OS-M synapses between M/Ts and OSNs, we stimulated ONL containing axonal terminals of OSNs and recorded excitatory postsynaptic currents (EPSCs) from M/Ts adjacent to the stimulation electrode with bicuculline included in the ACSF (Fig. 4g). The results showed that, there were no significant differences in the amplitude of OS-M EPSCs between SNCA-A53T and WT mice (Fig. 4h, i). For the PG-M synapses between M/Ts and periglomerular cells (PGCs), we stimulated the glomerular layer (GL) containing dendritic terminals of PGCs and recorded inhibitory postsynaptic currents (IPSCs) from M/Ts adjacent to the stimulation electrode with D-AP5 and CNQX in the ACSF (Fig. 4j). The results showed that, compared with WT mice, the amplitude of PG-M IPSCs decreased in SNCA-A53T mice (Fig. 4k, l). Finally, for the GC-M synapses between M/Ts and granule cells (GCs), we stimulated the granule cell layer (GCL) containing dendritic terminals of GCs and recorded IPSCs from M/Ts adjacent to the stimulation electrode with D-AP5 and CNQX in the ACSF (Fig. 4m). Consistent with the results in PG-M synapses, the IPSCs amplitude in GC-M synapses also decreased in SNCA-A53T mice (Fig. 4n, o). Collectively, these findings indicate that, at 6 months of age, SNCA-A53T mice showed an E/I imbalance of neural microcircuitry in OB, which might result from the impairment of GABAergic transmission in OB of these mice.

### Altered miniature inhibitory responses and hyperactivity of M/T cells in the OB of SNCA-A53T mice

**Fig. 5:**
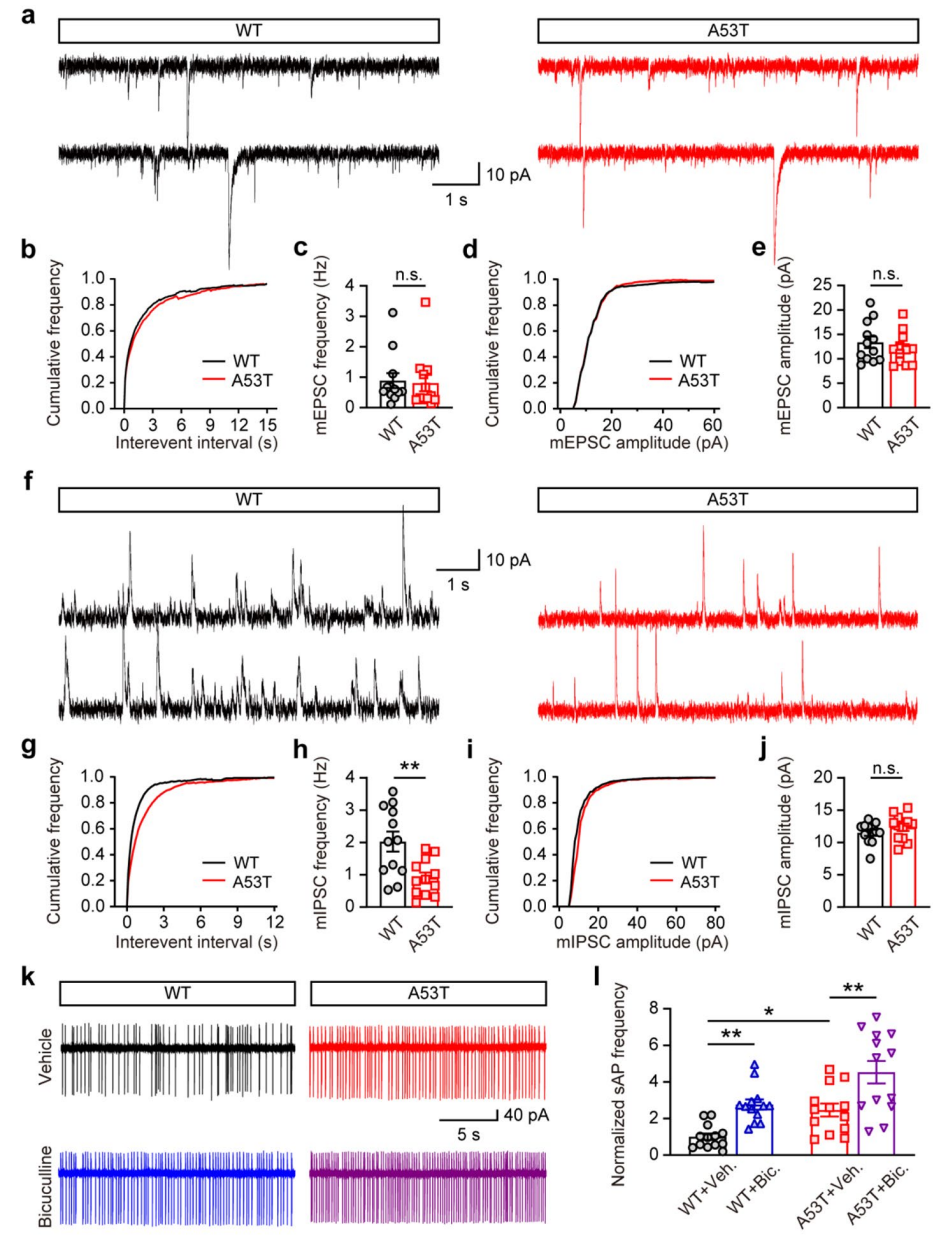
Decreased inhibitory synaptic responses and hyperactivity of M/Ts in OB of 6-month-old SNCA-A53T mice. **a** Representative images showing mEPSCs of M/Ts in OB. **b-c** Cumulative curves (**b**) and quantification (**c**) of mEPSCs frequency of M/Ts in OB of WT and A53T mice (n = 4 for each group). **d-e** Cumulative curves (**d**) and quantification (**e**) of mEPSCs amplitude of M/Ts in OB of WT and A53T mice (n = 4 for each group). **f** Representative images showing mIPSCs of M/Ts in OB. **g-h** Cumulative curves (**g**) and quantification (**h**) of mIPSCs frequency of M/Ts in OB of WT and A53T mice (n = 4 for each group). **i-j** Cumulative curves (**i**) and quantification (**j**) of mIPSCs amplitude of M/Ts in OB of WT and A53T mice (n = 4 for each group). **k** Representative images showing sAP of M/Ts in OB. **l** Quantitative analysis of normalized sAP frequency of M/Ts in OB of WT and A53T mice (n = 4 for each group). Data are presented as mean ± SEM. *p < 0.05; **p < 0.01; n.s., not significant

The E/I balance in the OB is determined by the glutamatergic input of M/Ts from OSNs and the broad feedback inhibition of M/Ts from interneurons [21]. To further investigate the underlying mechanisms of E/I imbalance in the OB, we conducted whole-cell recordings in M/Ts of OB slices. The results showed that, the amplitudes and frequencies of miniature EPSCs (mEPSCs) in M/Ts were similar between SNCA-A53T and WT mice (Fig. 5a-e), indicating that the excitatory synaptic transmission from OSNs to M/Ts was unchanged in SNCA-A53T mice. However, compared with WT mice, the frequency of miniature IPSCs (mIPSCs) of M/Ts was significantly reduced in SNCA-A53T mice, while the amplitudes of mIPSCs were similar between SNCA-A53T and WT mice (Fig. 5f-j), suggesting that the inhibitory synaptic transmission from interneurons to M/Ts was impaired in OB in SNCA-A53T mice.

In addition, we conducted cell-attached recordings in M/Ts of OB slices (Fig. 5k), and the results showed that, the firing rates of spontaneous action potentials (sAPs) in M/Ts were significantly increased in SNCA-A53T mice compared to WT mice, indicating hyperactivity of M/T cells. To reveal the underlying synaptic mechanisms of the hyperactivity of M/Ts, we further investigated whether it was related to the above aberrant GABAergic synaptic responses. The results showed that, the GABA_A_ receptor antagonist, bicuculline (10 µM), dramatically increased the firing rates of M/Ts in both SNCA-A53T and WT mice (Fig. 5l), however, the firing rates in WT mice increased by ~ 176.01%, while those in SNCA-A53T mice only increased by ~ 83.85% (Fig. 5l). Therefore, the hyperactivity of M/Ts is at least partly due to the impaired GABAergic synaptic transmission, which might subsequently contribute to the olfactory impairment in SNCA-A53T mice.

### Aberrant adult neurogenesis but unchanged interneuron quantity in the OB of SNCA-A53T mice

**Fig. 6:**
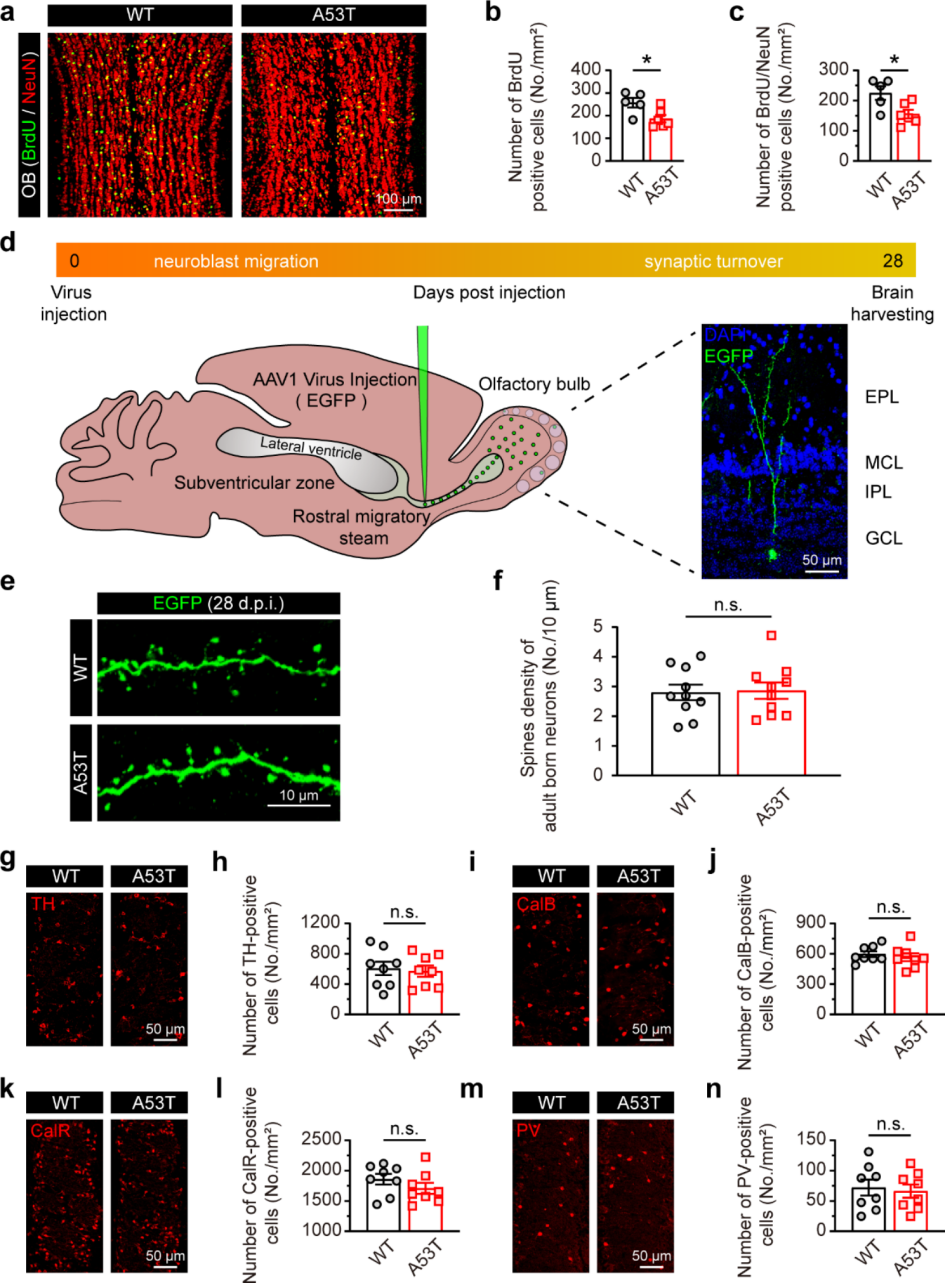
Altered adult neurogenesis in OB of 6-month-old SNCA-A53T mice. **a** Representative images showing BrdU and NeuN co-immunostaining of OB. **b-c** Quantitative analysis of BrdU-positive (**b**) and BrdU/NeuN double-positive (**c**) neurons in OB of WT and A53T mice (n = 3 for each group). **d** Schematic diagram illustrating labeling the adult-born neurons of OB by injection of the AAV1-CMV-EGFP into RMS. **e** Representative images showing the spines of adult-born neurons in OB. **f** Quantitative analysis of the spine density of adult-born neurons in OB of WT and A53T mice (n = 3 for each group). **g-n** Representative images and quantification of the TH-positive (**g, h**), CalB-positive (**i, j**), CalR-positive (**k, l**), and PV-positive (**m, n**) neurons in OB of WT and A53T mice (n = 3 for each group). Data are presented as mean ± SEM. *p < 0.05; n.s., not significant

The above results suggest that the GABAergic system dysfunction is essential for olfactory impairment in SNCA-A53T mice. Interneurons are considered the primary source of GABAergic inhibition, and they undergo a process of migration and differentiation originating from the subventricular zone (SVZ), making them a focus of investigation for adult neurogenesis in OB [22]. We further examined the adult neurogenesis in OB by BrdU incorporation. The immunostaining analysis revealed that, compared with WT mice, the BrdU-positive and BrdU/NeuN double-positive cells, i.e., adult-born cells and neurons, respectively, in the OB of SNCA-A53T mice were decreased (Fig. 6a-c). However, there was no significant difference in the spine density of adult-born neurons between SNCA-A53T and WT mice (Fig. 6d-f), indicating that the synaptic development of adult-born neurons was unimpaired in OB of SNCA-A53T mice. Additionally, the number of TH-, Calbindin (CalB)-, Calretinin (CalR)-, and Parvalbumin (PV)-positive interneurons was similar in OB between SNCA-A53T and WT mice (Fig. 6g-n), which may be attributed to the fact that adult-born neurons only account for a small fraction of interneurons. Therefore, these results suggest that the dysregulated GABAergic signaling was not due to the changes in interneuron quantity in 6-month-old SNCA-A53T mice.

### Altered GABA transport proteins in the OB of SNCA-A53T mice

**Fig. 7:**
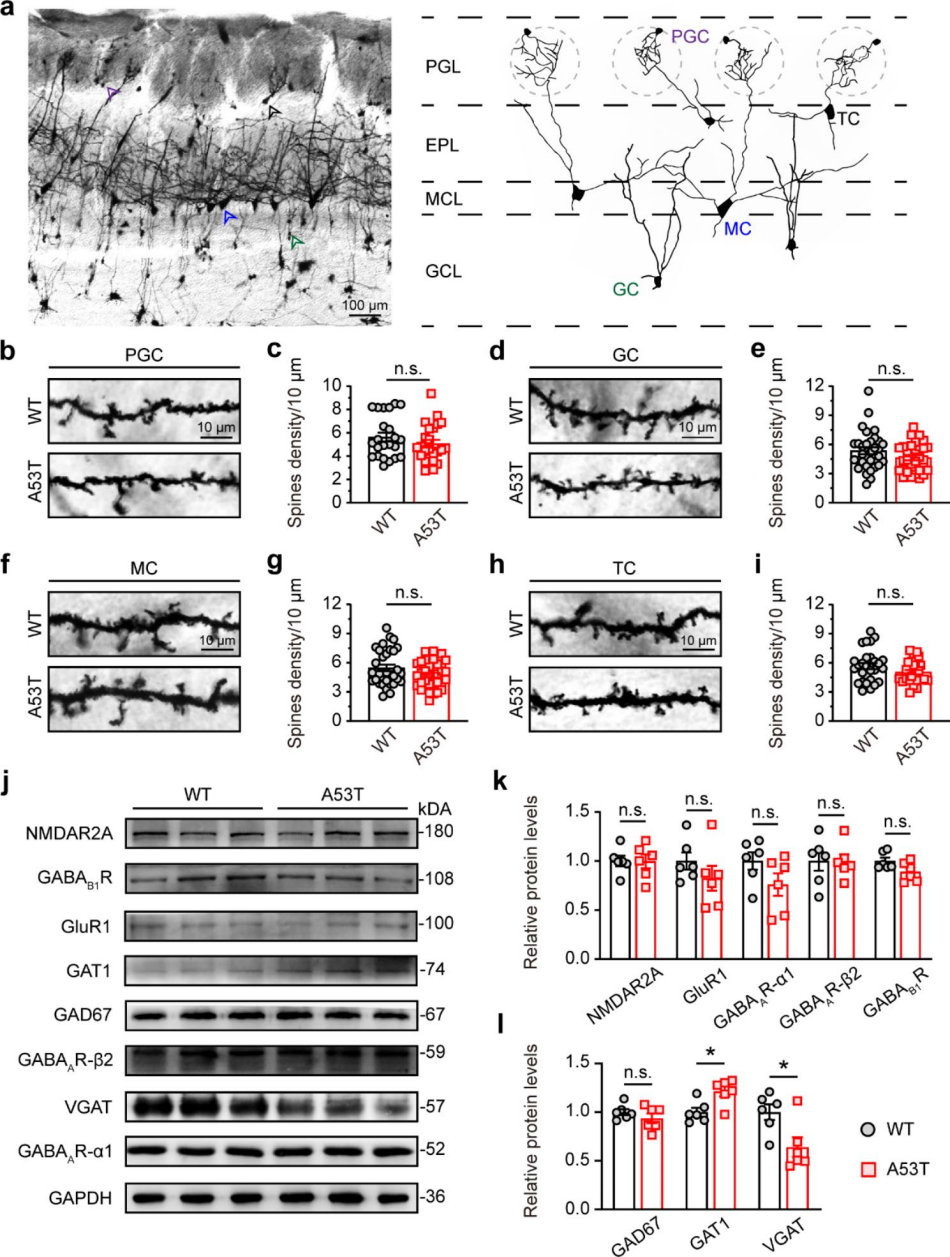
Aberrant GABA transport protein in OB of 6-month-old SNCA-A53T mice. **a** Representative Golgi staining image showing four kinds of neurons in different layers of OB. The purple arrow indicates PGC; the green arrow indicates GC; the blue arrow indicates MC; the black arrow indicates TC. **b-i** Representative Golgi staining images and quantification of spine density of PGC (**b, c**), GC (**d, e**), MC (**f, g**), and TC (**h, i**) in OB of WT and A53T mice (n = 4 for each group). **j-l** Representative immunoblotting images (**j**) and quantification (**k, l**) of the levels of NMDAR2A, GluR1, GABA_A_R-α1, GABA_A_R-β2, GABA_B1_R, GAT1, VGAT and GAD67 in OB of WT and A53T mice (n = 6 for each group). Data are presented as mean ± SEM. *p < 0.05; n.s., not significant

Previous studies have shown that α-synuclein accumulates in neuronal terminals and affects the synaptic structure and spine formation [23]. We further investigated the synaptic mechanisms underlying the E/I imbalance in the OB in SNCA-A53T mice. Using Golgi staining, we distinguished the mitral cells (MCs), tufted cells (TCs), PGCs, and GCs based on cell location and morphology (Fig. 7a). Histological analysis showed that, these four kinds of neurons displayed no significant difference in spine density between SNCA-A53T and WT mice (Fig. 7b-i), indicating no apparent morphological changes in spine development. We further examined the synaptic proteins by immunoblotting (Fig. 7j). The results showed that, compared with WT mice, the GAT1 protein level of OB was significantly increased in SNCA-A53T mice, while the vesicular GABA transporter (VGAT) protein level was significantly decreased (Fig. 7l), indicating that the GABA transport was damaged in SNCA-A53T mice. However, the protein levels of NMDAR2A, GluR1, GABA_A_R-α1, GABA_A_R-β2, GABA_B1_R and GAD67 were not significantly changed (Fig. 7k, l), indicating that the glutamate and GABA receptors, and the GABA synthesis were unaffected. Taken together, these results suggest that the dysregulated GABA transport may be responsible for the abnormal GABAergic signaling in the OB of SNCA-A53T mice.

### Rescued olfactory behavior and GABAergic responses of SNCA-A53T mice after TGB treatment

**Fig. 8:**
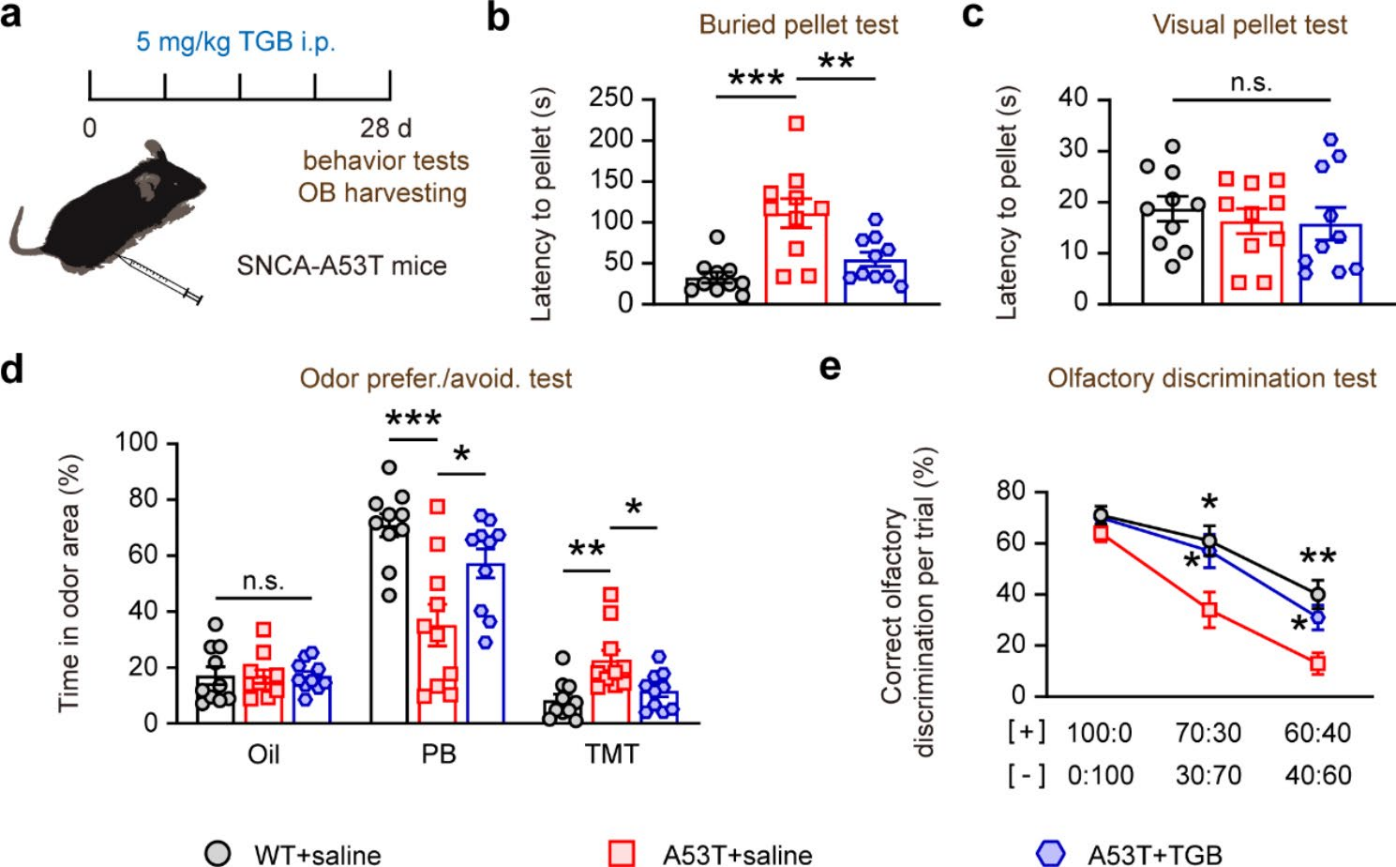
Improved olfactory behaviors of 6-month-old SNCA-A53T mice after administration of TGB. **a** Schematic diagram illustrating the experimental timeline of SNCA-A53T mice administrated with TGB. **b-c** Quantification of latency to find a food pellet buried (**b**) or on the surface (**c**) of bedding in WT and A53T mice treated with saline or TGB (n = 10 for each group). **d** Percentage of time spent sniffing the odor area containing oil, PB, and TMT in WT and A53T mice treated with saline or TGB (n = 10 for each group). **e** Percentage of correct olfactory discrimination per trial session in WT and A53T mice treated with saline or TGB (n = 10 for each group). Data are presented as mean ± SEM. *p < 0.05; **p < 0.01; ***p < 0.001; n.s., not significant

Since the GABAergic inhibition was significantly impaired and the GAT1 level was upregulated in SNCA-A53T mice, we further investigated whether enhancing the GABAergic inhibitory responses could ameliorate the olfactory dysfunction in SNCA-A53T mice. The SNCA-A53T mice were administrated with TGB, a selective inhibitor of GAT1 for 4 weeks, and olfactory behavioral tests were performed again. We found that, after daily intraperitoneal (i.p.) injection of TGB for 4 weeks, SNCA-A53T mice spent less time finding the hidden food pellet than vehicle-treated mice in the buried food pellet test (Fig. 8a, b), while all groups required a similar time to obtain the food pellet on the bedding in the visual food pellet test (Fig. 8c). Additionally, compared with the vehicle-treated mice, SNCA-A53T mice treated with TGB spent an extended time sniffing the filter paper containing PB, but spent less time investigating the filter paper containing TMT (Fig. 8d). Moreover, the percentage of correct olfactory discrimination responses per trial was increased in SNCA-A53T mice after TGB administration (Fig. 8e). These results indicate that intraperitoneal administration of TGB for 4 weeks could ameliorate the abnormal olfactory behavior in SNCA-A53T mice.

**Fig. 9:**
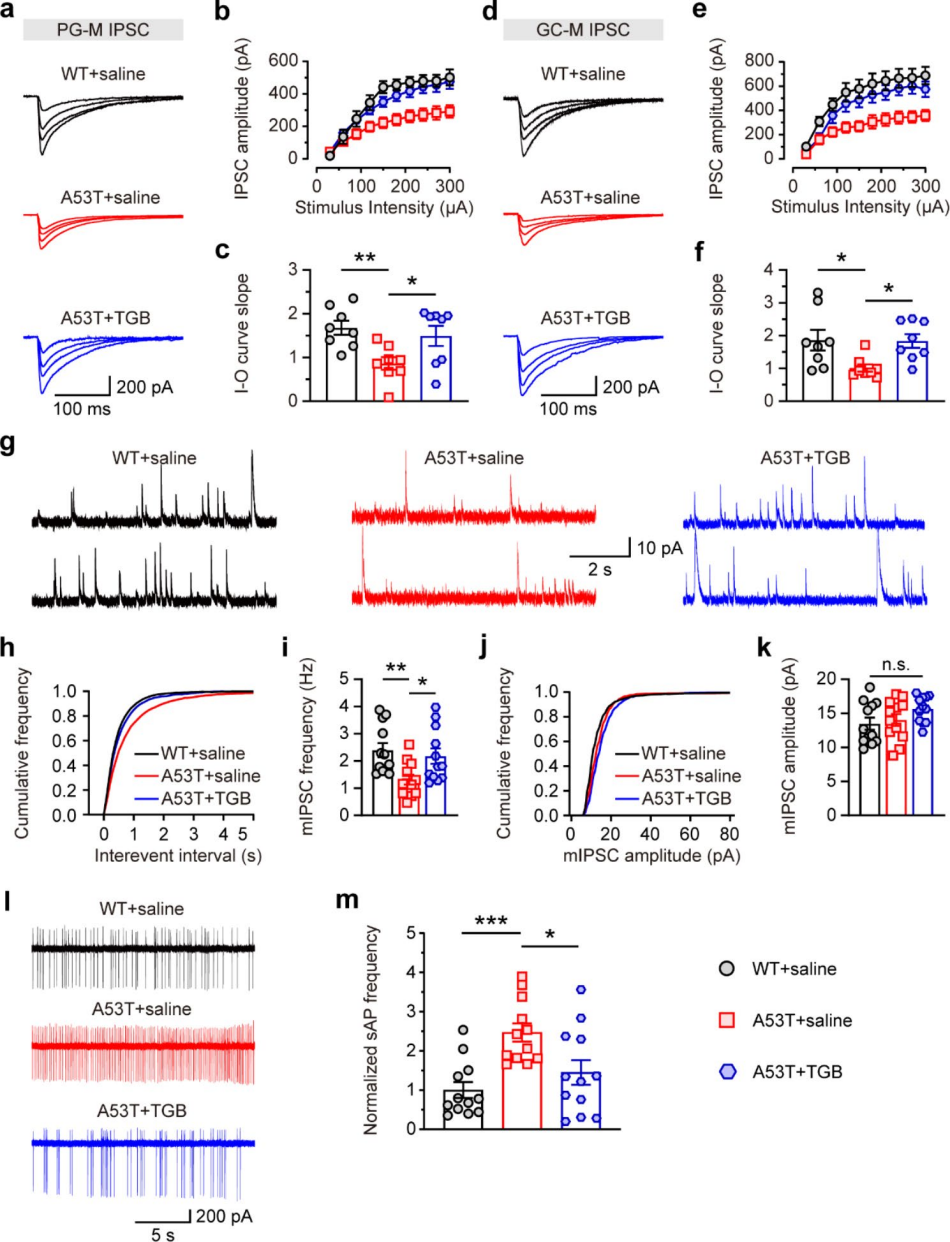
Enhanced inhibitory effects of M/Ts in OB of 6-month-old SNCA-A53T mice after administration of TGB. **a-b** Representative images (**a**) and input-output curves (**b**) of PG-M IPSCs of M/Ts in OB of WT and A53T mice treated with saline or TGB (n = 4 for each group). **c** Quantitative analysis of the slope of (**b**) in WT and A53T mice treated with saline or TGB (n = 4 for each group). **d-e** Representative images (**d**) and input-output curves (**e**) of GC-M IPSCs of M/Ts in OB of WT and A53T mice treated with saline or TGB (n = 4 for each group). **f** Quantitative analysis of the slope of (**e**) in WT and A53T mice treated with saline or TGB (n = 4 for each group). **g** Representative images showing mIPSCs of M/Ts in OB. **h-i** Cumulative curves (**h**) and quantification (**i**) of mIPSCs frequency of M/Ts in OB of WT and A53T mice treated with saline or TGB (n = 4 for each group). **j-k** Cumulative curves (**j**) and quantification (**k**) of mIPSCs amplitude of M/Ts in OB of WT and A53T mice treated with saline or TGB (n = 4 for each group). **l-m** Representative images (**l**) and quantification (**m**) of normalized sAP frequency of M/Ts in OB of WT and A53T mice treated with saline or TGB (n = 4 for each group). Data are presented as mean ± SEM. *p < 0.05; **p < 0.01; ***p < 0.001; n.s., not significant

Next, we further investigated the effects of TGB on inhibitory synaptic transmission in the OB of SNCA-A53T mice. Patch-clamp analysis showed that administration of TGB could increase the amplitude of PG-M and GC-M IPSCs of M/Ts in the OB of SNCA-A53T mice (Fig. 9a-f). We also observed that TGB administration could increase the frequency of mIPSCs of M/Ts in the OB of SNCA-A53T mice (Fig. 9g-k), indicating a rescued effect of TGB on inhibitory synaptic transmission in OB. Additionally, we found that the sAP firing rates of M/Ts were significantly decreased in OB slices from the TGB-treated SNCA-A53T mice (Fig. 9l, m). These results suggest that TGB administration could ameliorate the hyperactivity of M/Ts and abnormal neural microcircuitry in OB in SNCA-A53T mice.

## Discussion

**Fig. 10:**
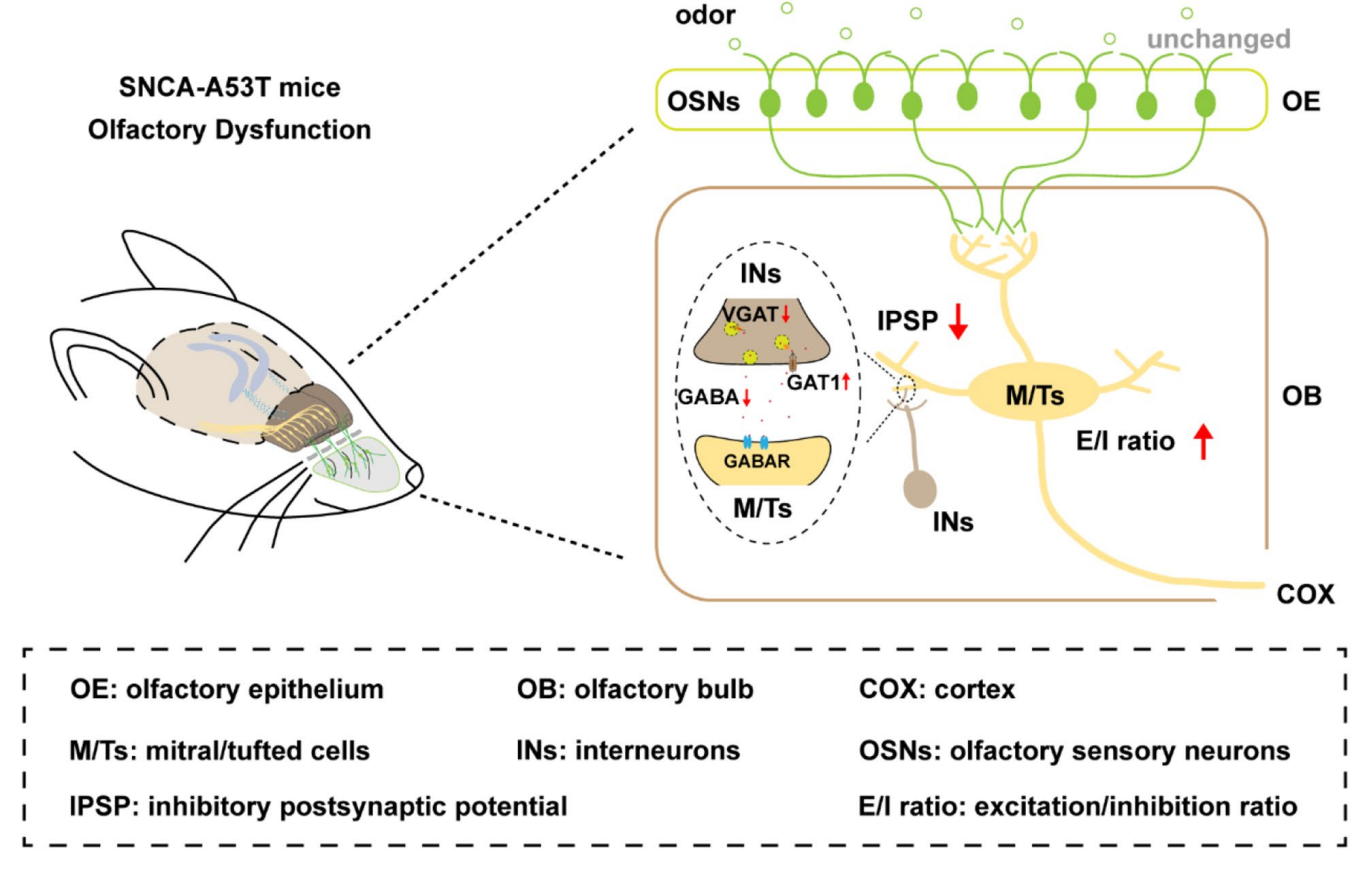
Schematic illustration of the local neural microcircuit mechanisms underlying olfactory dysfunction in SNCA-A53T mice. At 6 months of age, SNCA-A53T mice exhibited unchanged OSNs and glutamatergic innervation from OE to OB, but displayed impaired GABAergic inhibition of M/Ts from multiple interneurons in different layers of OB, which was attributed to the aberrant GAT1 and VGAT levels and GABAergic transmission. Together, these alterations led to the hyperactivity of M/Ts, the E/I imbalance in OB and subsequent olfactory dysfunction

Olfactory dysfunction is a cardinal prodromal symptom of various neurodegenerative disorders, including PD, and the olfactory pathway is especially vulnerable to α-synuclein pathology [4, 20]. However, the precise synaptic mechanisms underlying neural microcircuit disharmony in the primary olfactory pathway at the early stage of PD remain incompletely understood. Herein, we probed the spatial distribution characteristics of α-synuclein in the primary olfactory pathway and investigated the synaptic mechanisms of OB in SNCA-A53T mice. Our results revealed that 6-month-old SNCA-A53T mice displayed anomalous olfactory behaviors linked to E/I imbalance, specifically, dysregulated GABAergic signaling in OB (Fig. 10). Strikingly, typical pathological changes in OB occurred earlier than those in OE, SNc, and STR. Significantly, we demonstrated that bolstering GABAergic signaling with TGB ameliorated abnormal olfactory behavior, impaired GABAergic synaptic transmission and hyperactivity of M/Ts in OB. Hence, GABAergic dysfunction could represent a promising therapeutic target for olfactory impairment in early-stage PD.

Mounting evidence suggests that the presence of α-synuclein deposits in the olfactory pathway is an early indicator of PD pathologies in rodents and patients. In particular, α-synuclein BAC transgenic mice, a model of prodromal PD, could exhibit hyposmia with native α-synuclein expression patterns in OB [24]. Similarly, transgenic mice overexpressing α-synuclein with A53T or A30P mutants displayed olfactory dysfunction and α-synuclein pathology in OB before the onset of motor symptoms [11, 20]. In PD patients, α-synuclein was found throughout the OB [25–27]. Furthermore, RT-QuIC analysis suggested α-synuclein seeded in the olfactory mucosa of PD patients, especially in the early stages of PD [17–19]. Despite these findings, controversy remains regarding the initial site of α-synuclein deposits in the primary olfactory pathway of PD, which could affect the early diagnosis and treatment. According to Braak staging, α-synuclein is triggered in the OB and then spreads to other brain areas [10]. Nevertheless, recent reports suggest that α-synuclein may initially seed in the olfactory mucosa [28]. To shed light on this issue, we investigated the distribution of α-synuclein in the OB and OE, and our results showed that α-synuclein significantly increased and accumulated in OB but not in OE of 6-month-old SNCA-A53T mice. Moreover, SNCA-A53T mice exhibited apparent neural microcircuitry disharmony in OB but no significant changes in cytoarchitecture and function of OE. Therefore, it is possible that α-synuclein initially seeds in the OB and then spreads to the OE or other brain areas. However, further studies remain required in other models and patients to determine the initial site of α-synuclein deposits in the primary olfactory pathway.

As noted above, OB is one of the earliest brain regions affected by α-synuclein deposition, and serves as the primary olfactory information integration center [10, 29]. The central output neurons of the OB are the M/Ts, which receive excitatory input from OE as well as inward inhibitory feedback, and thus reflect the E/I balance. Extensive researches have highlighted the critical role played by the E/I balance in neural function and memory [21, 30–33]. A recent study also indicated that α-synuclein overexpression restricted to the OB could directly induce an impairment of GABAergic synaptic transmission between GCs and M/Ts [16], but the exact characteristics of the neural microcircuitry disharmony in OB are not yet clear. In this study, we observed no significant change in excitatory innervation from OE to OB, accompanied by almost intact cytoarchitecture in OE. Besides, there was decreased GABAergic inhibition from multiple interneurons in different layers of OB, along with hyperactivity of M/Ts, which resulted in E/I imbalance. These findings are supported by previous reports that hypoactivity of GCs induced by α-synuclein is responsible for GABAergic dysfunction of M/Ts in OB [16]. Additionally, we further determined that the weakness of inward GABAergic inhibition was at least partly responsible for the hyperactivity of M/Ts. Consistent with this, it is known that the GCL and internal plexiform layer (IPL) in OB mainly contain diverse interneurons, and α-synuclein is expressed predominantly in neuronal fibers of the GCL and IPL of the OB at the early stage of PD [20, 34, 35]. These researches imply that, the GABAergic dysfunction in OB is strongly related to α-synuclein pathology and might be the direct factor of olfactory impairments at the early stage of PD.

We further elucidated the synaptic transmission characteristics involved in the E/I imbalance. Patch-clamp recordings revealed an unchanged amplitude but a reduced frequency of mIPSC. Typically, changes in the amplitude of mini postsynaptic currents represent alterations in postsynaptic effects, whereas frequency changes are largely due to the release of presynaptic neurotransmitters. It has been reported that α-synuclein deposits can induce structural and functional damage in the presynaptic area [36–39]. We thus assessed the factors influencing presynaptic effects, including the overall interneurons and the complex synaptic proteins. Firstly, the regeneration of interneurons in the OB occurs over a lifetime, and migrating neuroblasts from the SVZ differentiate into mature interneurons in the OB [22, 40]. We observed fewer adult-born neurons in the OB, supported by the similar pathologic changes in A30P-transgene and MPTP inducible mice [20, 41, 42]. However, the impaired adult neurogenesis is insufficient to affect the total number of multiple interneurons at the testing time. One possibility is that adult-born neurons only account for a small fraction of interneurons, roughly 1% of the total OB neurons [43, 44]. These findings suggest that this testing time might be at the very early stage of PD. Additionally, we found upregulated GAT1 and downregulated VGAT levels in the OB of SNCA-A53T mice. However, other synapse-related proteins, including glutaminergic receptors, GABAergic receptors and GABA synthetase, were not altered in SNCA-A53T mice. These findings suggest that excessive reabsorption of GABA from the synaptic cleft and reduced GABA loading in synaptic vesicles may result in insufficient GABA levels in the synaptic cleft. Meanwhile, consistent with our findings, proteomic analysis of OB in A30P-transgene mice also revealed that α-synuclein regulated synaptic transmission by perturbing proteins involved in synaptic exo- and endocytosis pathways [20]. In addition, a recent study showed that, α-synuclein could interact with microtubule beta-III to form a toxic complex, which might regulate the release of neurotransmitters, such as GABA, in interneurons [45]. Thus, these findings suggest a dysregulated neurotransmitter transport in the presynaptic terminal at a very early stage of PD progression. Furthermore, indirect centrifugal innervations from the higher olfactory cortex might also be involved in the presynaptic effects [46, 47], which requires further investigation.

Growing evidence suggests that there are distinct olfactory and gastrointestinal pathologies in the early stage of PD, which can significantly impact social functioning [2, 3, 48–50]. However, early treatments for these pathologies in PD are rare. Recent studies have shown that regulating GABAergic signaling can affect neural networks [51–53]. Given the upregulated expression of GAT1 in OB of SNCA-A53T mice, we selected a potent GAT1 inhibitor, TGB, which is an anticonvulsant widely exploited in the clinic [54, 55], to be intraperitoneally administered to mice. Our results revealed that TGB could reduce the hyperactivity of M/Ts, regulate inhibitory neural microcircuit in OB, and improve olfactory function in SNCA-A53T mice. These findings are consistent with several studies showing that enhancing GABAergic inhibition could rescue olfactory impairments in mice with Alzheimer’s disease (AD) [51, 56]. Therefore, these results suggest that GABAergic signaling plays a pivotal role in neurodegenerative diseases such as PD and AD, and may be a potential target for the treatment of early-stage PD.

## Conclusions

Our findings provide novel insight into the initial site of α-synuclein and potential local neural microcircuit mechanisms of the primary olfactory pathway underlying olfactory dysfunction at the early stage of PD. More importantly, enhancing GABAergic signaling by TGB can regulate the inhibitory neural microcircuit of OB and improve olfactory function in the PD mouse model. Therefore, our studies highlight the effect of aberrant GABAergic signaling in OB on early diagnosis and provide a potential therapeutic strategy against early-stage PD.

## Methods

### Animals

All experimental procedures were approved by the Xiamen University Animal Care and Use Committee. Heterozygous offspring of the overexpressed A53T mutant mice (line G2-3, B6.Cg-Tg [Prnp-SNCA*A53T] 23 Mkle/J, Jackson Laboratories, stock no. 006823, Bar Harbor, ME, USA) were used for the study. Animals were maintained in cages under 12-h light and dark cycles, with free access to food and water. International and Xiamen University’s ethical standards were followed, and an approximately equal number of male and female mice were included in the experiments.

### Intraperitoneal and viral injections

TGB (Meilun) was dissolved in sterile saline and stored at -4 °C in advance. To avoid irreversible damage to the OB, TGB or saline was i.p. administered at a dose of 5 mg/kg once daily for 4 weeks, indicated as a long-term treatment.

5-bromo-2’-deoxyuridine (BrdU, Aladdin Biochemical Technology) was i.p. administered (50 mg kg^− 1^ body weight) once daily for 5 consecutive days to assess the proliferation of adult-born neurons in OB as described in the protocol [57]. And the mice were killed with deep anesthesia 32 days after the first BrdU injection.

Adeno-associated virus (AAV1) encoding EGFP under the control of CMV promoter (Han Heng biotechnology) was used to label adult-born neurons migrating from SVZ to OB as previously reported [58]. The mice were anesthetized deeply and their pinch withdrawal reflex was monitored to assess the depth of anesthesia. The skull of the mice was carefully exposed and opened with a 2–3 mm round hole by a dental drill. The surface of the skull and brain was cleaned with sterile saline. Stereotactic bilateral infusion of the AAV1 (500 nL) into the RMS (coordinates relative to Bregma: AP, -3.3 mm; ML, ± 0.8 mm; DV, -2.9 mm) was conducted. After the surgery, the mice recovered for 28 days until imaging.

### Buried food pellet test

The buried food pellet test was performed to evaluate odor detection ability based on the protocols [59]. The mice were deprived of food but had free access to water for 24 h prior to the experiment. The mice were placed into a clean test cage for 10 min to habituate before the test. Then the mice were transferred into the same test cage containing 3-cm-deep bedding with an approximately 400-mg food pellet buried 1 cm beneath the bedding randomly in a corner. The mice were maintained a constant distance from the hidden food and given 300 s to locate the buried pellet. The latency to locate the food pellet was measured. If the mouse failed to find the buried pellet within 300 s, the test was stopped, and the latency score was 300 s. As a control, there was a visual food pellet test. The mice were allowed to locate the food pellet on top of the bedding under the same conditions. The latency for finding the visual food pellet was defined as the time between placing the mouse in the cage and when it touched the food pellet with its forepaws or nose.

### Olfactory preference/avoidance test

The olfactory preference/avoidance test was carried out to evaluate the odor detection ability and native odor responses as previously reported with minor modifications [16]. PB was used as a fond odor in this test, and TMT, a compound derived from fox feces that induces innate fear in rodents was regarded as an aversive odor. The mice were allowed to be transferred to an empty test cage for 10 min acclimation. In the control odor testing, 20 µL mineral oil was applied to the filter paper in the cage to exclude the effect of filter paper and mineral oil. Then, 1 g peanut butter was mixed with 50 µL mineral oil, indicated as PB solution. In addition, 50 µL TMT (dissolved in mineral oil at 40% v/v dilution) was described as a TMT solution. 20 µL PB or TMT solution was applied to the filter paper, respectively. The mice were allowed to explore freely for 5 min to sniff the filter paper containing odor solution. The time spent sniffing the filter paper containing odor solution was recorded. The sniffing was defined as the distance between its nose and the center of filter paper within 1 cm.

### Olfactory discrimination test

The olfactory discrimination test was modified from previous methods to evaluate the olfactory discrimination ability by associating olfaction with taste aversion [59]. Mice were separated into individual cages and deprived of water but had free access to food for 24 h before the experiment. In the training stage, each mouse was placed into a clean cage to associate mango scents with tasty drinks, and almond scents with bitter drinks. The combination of distilled water and mango extract (Mgo), defined as [+], served as a reward for the response. A sterile 35 × 10-mm dish containing a mixture of 10 µL with distilled water and Mgo was placed into the cage to allow the mice to habituate to the Mgo scents. Afterward, the Mgo volume was steadily increased to 1, 2.5, 4, 5.5, 7, and 8.5 µL, and each mouse was allowed to discover [+] for 2 min with a 30 s interval of trials. After the Mgo trials, we provided the mice with 8.5 µL almond extract (ALM) mixed with 1.5 µL 1% denatonium benzoate (DB) solution, which was regarded as [-]. In order to make the mice associate a bitter taste with ALM, we repeated this trial four times. Because of the bitter nature of DB, the mice were disgusted with the [-] solution and avoided selecting it in the following tests. In the testing stage, both [+] and [-] solutions were placed in the testing cage with two dishes and mixed in different fractions. One dish mainly contained [+] solution, and the other dish primarily held [-] solution. For example, the 70:30 ratio of the odor solution indicated that the composition ratio of Mgo in distilled water to ALM in 1% DB in one dish was 70:30. The ratio in the other dish was 30:70. One mouse only chose the [+] solution within 30 s, which was regarded as a success. If the mice chose [-] solution or both [+] and [-] within 30 s, the behaviors were considered failures. And if one mouse did not choose anyone, it was excluded. A total of ten trials were conducted in each mouse, and the percentage of successful olfactory discrimination was measured.

### Rotarod test

The rotarod test was conducted to evaluate the motor coordination of the mice. In training, each mouse was placed on the rotating rod for 180 s once daily for 3 consecutive days, and the rod was constantly rotated at 20 rpm. If the mice fell into the ground, they must return to the rotating rod until finishing 180-s training. In the testing, the mice were placed on the rotating rods (20 rpm), and the time taken for the mice to fall from the rotating rods was recorded. If the mice were kept on the rotating rods for 180 s, the time score was 180 s. This trial was repeated three times, and the time score was averaged.

### Pole test

The pole test was used to detect the motor coordination ability of mice. The mice were placed on the top of a rough-surfaced pole (55 cm height, 1 cm diameter). The time to climb down the pole was recorded. Trails were excluded if the mice slid down or jumped out of the pole. This trial was repeated three times, and the time score was averaged.

### Hanging wire test

The hanging wire test was used to assess the neuromuscular strength of the paws of the mice. The mice were suspended on the middle of a horizontal rod (30 cm long, 2 mm diameter, between two 50 cm high poles), and the latency of suspension was measured within 30 s. We gave the mice score according to the latency of suspension: 0 ~ 4 s (score as 0), 5 ~ 9 s (score as 1), 10 ~ 14 s (score as 2), 15 ~ 19 s (score as 3), 20 ~ 24 s (score as 4), 25 ~ 29 s (score as 5), 30 s (score as 6). If the mice climbed to the high poles along the rod, the score was defined as 6. This trial was repeated three times, and the score was averaged.

### Tissue Preparation

After behavioral tests, the mice were sacrificed under deep anesthesia and perfused with 0.01 M phosphate-buffered saline (PBS, pH 7.4) and 4% paraformaldehyde in 0.1 M PB solution (pH 7.4). The OE and brain sections, including OB, STR, and SNc, were taken, dehydrated and waxed step by step. One set of mice was cut coronally using a manual rotary microtome and stored at 4℃ for Nissl staining and immunohistochemical staining; the other set was cut by a freezing microtome and held at -20℃ for immunofluorescence staining. Another set of mice from each group was used for western blotting, electrophysiology, and Golgi staining.

### Nissl staining

The OE slices were rinsed three times with 0.01 M PBS, dewaxed at 65℃ in a drying cabinet, and rinsed three times again. The slices were defatted in 75% ethanol at 37℃ for 2 h, stained with 0.1% cresyl violet solution for 10 min, and rinsed with water. Then the slices were sequentially incubated with ethanol and xylene. The slides were covered with permanent cover slips. An Olympus microscope captured images.

### Immunohistochemical staining

The slices, including OE, OB, STR, and SNc, were rinsed three times with 0.01 M PBS, dewaxed at 65℃ in a drying cabinet, and rinsed again. And the slices were incubated with 3% H_2_O_2_ for 30 min, blocked with 10% goat serum in PBS, and then incubated with primary antibodies against α-synuclein (BD Transduction Laboratories, 1:200) and TH (Sigma, 1:500) overnight at 4℃, respectively. The slices were then rinsed with PBS and incubated with secondary antibodies conjugated to HRP (Invitrogen Technologies, 1:1000) for 2 h at room temperature. The pieces were subsequently stained with DAB, mounted on gelatin-coated glass slides, and cover-slipped. The pieces were imaged with an Olympus microscope. The images were quantitatively analyzed by Image J software.

### Immunofluorescence staining

The OE and OB slices stored at -20℃ were washed three times with 0.01 M PBS, blocked with 10% goat serum in PBS, and incubated with primary antibodies against OMP (Abcam, 1:800), BrdU (Abcam, 1:500), NeuN (Abcam, 1:1000), TH (Sigma, 1:500), CalR (Santa Cruz, 1:300), CalB (Cell Signaling Technology, 1:500), PV (Swant, 1:1000) and GFP (Abcam, 1:100) overnight at 4℃, respectively. The slices were then rinsed with PBS and incubated with secondary antibodies conjugated to Alexa Fluor 488 (Invitrogen Technologies, 1:1000) and Alexa Flour 594 (Invitrogen Technologies, 1:1000) for 1 h at 37℃ in a drying cabinet. The slices were subsequently rinsed with PBS, mounted on gelatin-coated glass slides with fluorescent sealant, and cover-slipped. The slices were imaged with a laser-scanning confocal microscope (Olympus FV1000, Japan). The images were quantitatively analyzed by Image J software.

### Western blotting

The OB and OE sections were removed, dissected with a surgical blade, and homogenized in lysis buffer with complete protease and phosphatase inhibitors on ice. Tissue homogenates were collected, and protein quantitation was performed by the BCA method. Equal protein amounts of denatured samples were subjected to sodium dodecyl sulfate-polyacrylamide gel electrophoresis on 8 ~ 12% polyacrylamide gels and then transferred to nitrocellulose membranes. The membranes were incubated in 3% bovine serum albumin at room temperature for 2 h before being probed with primary and secondary antibodies. The primary antibodies used were as follows: anti-α-synuclein (BD Transduction Laboratories, 1:500), anti-OMP (Abcam, 1:1000), anti-NMDAR2A (Cell Signaling Technology, 1:500), anti-GABA_B1_R (Proteintech, 1:500), anti-GluR1 (Proteintech, 1:500), anti-GAT1 (Proteintech, 1:500), anti-GAD67 (Proteintech, 1:1000), anti-GABA_A_R-β2 (Proteintech, 1:1000), anti-VGAT (Proteintech, 1:500), anti-GABA_A_R-α1 (Proteintech, 1:1000) and anti-GAPDH (Proteintech, 1:5000). Proteins were visualized using the enhanced chemiluminescence method. The scanned images were quantified by Image J software. Specific bands were then quantified and normalized to the GAPDH loading control for each lane and each blot.

### Electrophysiology

The electrophysiology methods were modified from previous reports [51, 60, 61]. The OB horizontal slices (350 μm) were prepared by a vibratome (VT1000S, Leica) for patch clamp recording in an ice-cold oxygenated (95% O_2_, 5% CO_2_) cutting solution (pH 7.2 ~ 7.4) comprised of (in mmol/L) N-methyl-D-glucamine (93), HCl (93), KCl (2.5), NaH_2_PO_4_ (1.2), NaHCO_3_ (30), HEPES (20), glucose (25), (+)-sodium L-ascorbate (5), thiourea (2), sodium pyruvate (3), CaCl_2_ (0.5) and MgSO_4_ (10). After the slices were incubated in the cutting solution for 10 min at 32 ± 0.5 °C, the slices were rapidly transferred into the oxygenated (95% O_2_, 5% CO_2_) incubating solution (pH 7.2 ~ 7.4) for 1 h at room temperature. The incubating solution included (in mmol/L) NaCl (92), KCl (2.5), NaH_2_PO_4_ (1.2), NaHCO_3_ (30), HEPES (20), glucose (25), (+)-sodium Lascorbate (5), thiourea (2), sodium pyruvate (3), CaCl_2_ (2) and MgSO_4_ (2). The artificial cerebral spinal fluid (aCSF, pH 7.2 ~ 7.4), which comprised (in mmol/L) NaCl (124), KCl (2.5), CaCl_2_ (2), NaH_2_PO_4_ (1.2), NaHCO_3_ (24), MgSO_4_ (2), glucose (12.5) and HEPES (5), was oxygenated (95% O_2_, 5% CO_2_) throughout the tests and used for the following recording.

For field potential recording, fEPSPs were evoked with a two-concentrical bipolar stimulating electrode (FHC, Inc) and recorded with pipettes (1 ~ 2 MΩ) filled with aCSF under current-clamp mode. The stimuli consisted of monophasic 100-µs pulses of constant currents at 0.05 Hz. To distinguish the mixed fEPSPs and pure fEPSPs, picrotoxin (Sigma, 100 µM) was added to the aCSF during recording. For the whole-cell recording, eEPSC (holding at -70 mV) and eIPSC (holding at -70 mV) were evoked with the above stimulating electrode under voltage-clamp mode and recorded with pipettes (3 ~ 5 MΩ) filled with internal solution (pH 7.2 ~ 7.4) containing (in mmol/L) CsCH_3_SO_3_ (140), MgCl_2_ (2), TEA-Cl (5), HEPES (10), EGTA (1), Mg-ATP (2.5), Na_2_-GTP (0.3) and QX314 (5). The stimuli were similar to fEPSCs. mEPSC (holding at -70 mV) and mIPSC (holding at 0 mV) were recorded under voltage-clamp mode with pipettes (3 ~ 5 MΩ) filled with internal solution containing (in mmol/L) CsCH_3_SO_3_ (140), MgCl_2_ (2), TEA-Cl (5), HEPES (10), EGTA (1), Mg-ATP (2.5) and Na_2_-GTP (0.3). And for mEPSC and mIPSC recordings, 1 µM tetrodotoxin (TTX) was added to the aCSF. For mEPSC and eEPSC recordings, the aCSF was supplemented with bicuculline (20 µM), while for mIPSC and eIPSC recordings, 50 µM DL-2-amino-5-phosphonovaleric acid and 20 µM CNQX were required to be added to the aCSF. For the cell-attached recording, sAP was recorded under voltage-clamp mode with pipettes (3 ~ 5 MΩ) filled with aCSF. Data were filtered at a low-pass filter (2 kHz), sampled at 10 kHz by the Axon MultiClamp 700B amplifier (Molecular Devices) and digitized by pClamp10 (Molecular Devices). The quantitative analysis was conducted by Mini Analysis (Synaptosft) and Clampfit10 software (Molecular Devices).

### Golgi staining

The spine density of different neurons was assessed by a rapid Golgi staining kit (FD Neurotechnologies). Briefly, the brains were collected and stored in the mixed Golgi solution in the dark for 21 days. The brains were transferred to a cryoprotection solution for 3 days and sectioned horizontally with a vibratome (150 μm). The sections were placed on clean gelatin-coated microscope slides and stained with a certain solution. Then, the sections were rinsed with distilled water and dehydrated with successive baths of alcohol and mounted on glass cover-slips. The sections were imaged with a Slice Scanner (FV1000, Olympus). The images were quantitatively analyzed by Image J software. Spines on the secondary dendrites of PGCs, GCs, MCs, and TCs were involved in the analysis.

### Statistics

The statistical analysis of the data was performed using Prism 9.4.1 software (GraphPad). Data were subjected to unpaired t test or nonparametric Kolmogorov-Smirnov test for two-group comparisons, and one-way analysis of variance (ANOVA) followed by the Bonferroni post-hoc test for multiple comparisons. And n represents the number of animals in the figure legends. The significance level was set at P < 0.05. All data are expressed as the mean ± standard error of the mean (SEM).

## Electronic supplementary material

Below is the link to the electronic supplementary material.

Supplementary Material 1

## Data Availability

The additional data and materials during the current study are available from the corresponding author on reasonable request.

## Abbreviations

PD: Parkinson’s disease
OE: Olfactory epithelium
OB: Olfactory bulb
OSNs: Olfactory sensory neurons
M/Ts: Mitral/tufted cells
SNc: Substantia nigra pars compacta
RT-QuIC: Real-time quaking-induced conversion
TH: Tyrosine hydroxylase
STR: Striatum
E/I: Excitation/inhibition
TGB: Tiagabine
GAT1: GABA transporter 1
WT: Wild-type
PB: Peanut butter
TMT: 2,4,5-trimethylthiazole
OMP: Olfactory marker protein
PGL: Periglomerular layer
ONL: olfactory nerve layer
fEPSPs: Field excitatory postsynaptic potentials
EPL: External plexiform layer
fIPSPs: Field inhibitory postsynaptic potentials
EPSCs: Excitatory postsynaptic currents
PGCs: Periglomerular cells
GL: Glomerular layer
IPSCs: Inhibitory postsynaptic currents
GCs: Granule cells
GCL: Granule cell layer
sAPs: Spontaneous action potentials
SVZ: Subventricular zone
CalB: Calbindin
CalR: Calretinin
PV: Parvalbumin
MCs: Mitral cells
TCs: Tufted cells
VGAT: Vesicular GABA transporter
i.p.: Intraperitoneal
IPL: Internal plexiform layer
AD: Alzheimer’s disease
BrdU: 5-bromo-2’-deoxyuridine.
