# Supplementary material for: Amelioration of olfactory dysfunction in a mouse model of Parkinson’s disease via enhancing GABAergic signaling
Source: Cell Biosci. 2023 Jun 3;13:101. doi: 10.1186/s13578-023-01049-9 (PMC10239587; doi:10.1186/s13578-023-01049-9)
Supplement: Supplementary file 1 — Supplementary Material 1 [file 13578_2023_1049_MOESM1_ESM.docx]

**Additional file 1**


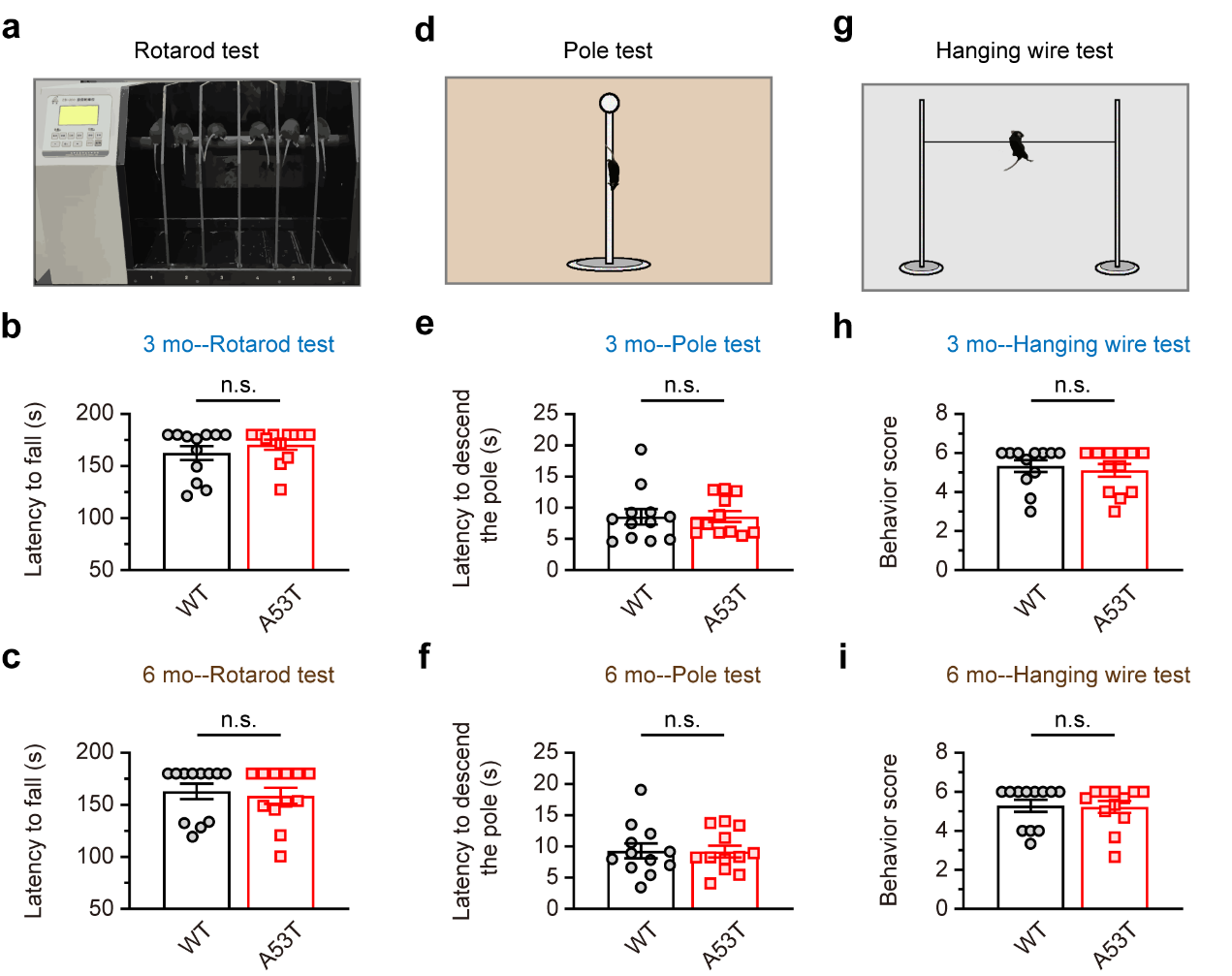
**Fig. S1** Unchanged motor ability of SNCA-A53T mice. **a** Schematic diagram of rotarod test. **b-c** Latency to fall to the ground in 3-month-old (**b**) and 6-month-old (**c**) WT and A53T mice (n=12 for each group). **d** Schematic diagram of pole test. **e-f** Latency to descend the pole in 3-month-old (**e**) and 6-month-old (**f**) WT and A53T mice (n=12 for each group). **g** Schematic diagram of hanging wire test. **h-i** Behavioral score of the hanging wire test in 3-month-old (**h**) and 6-month-old (**i**) WT and A53T mice (n=12 for each group). Data are presented as mean ± SEM. n.s., not significant.


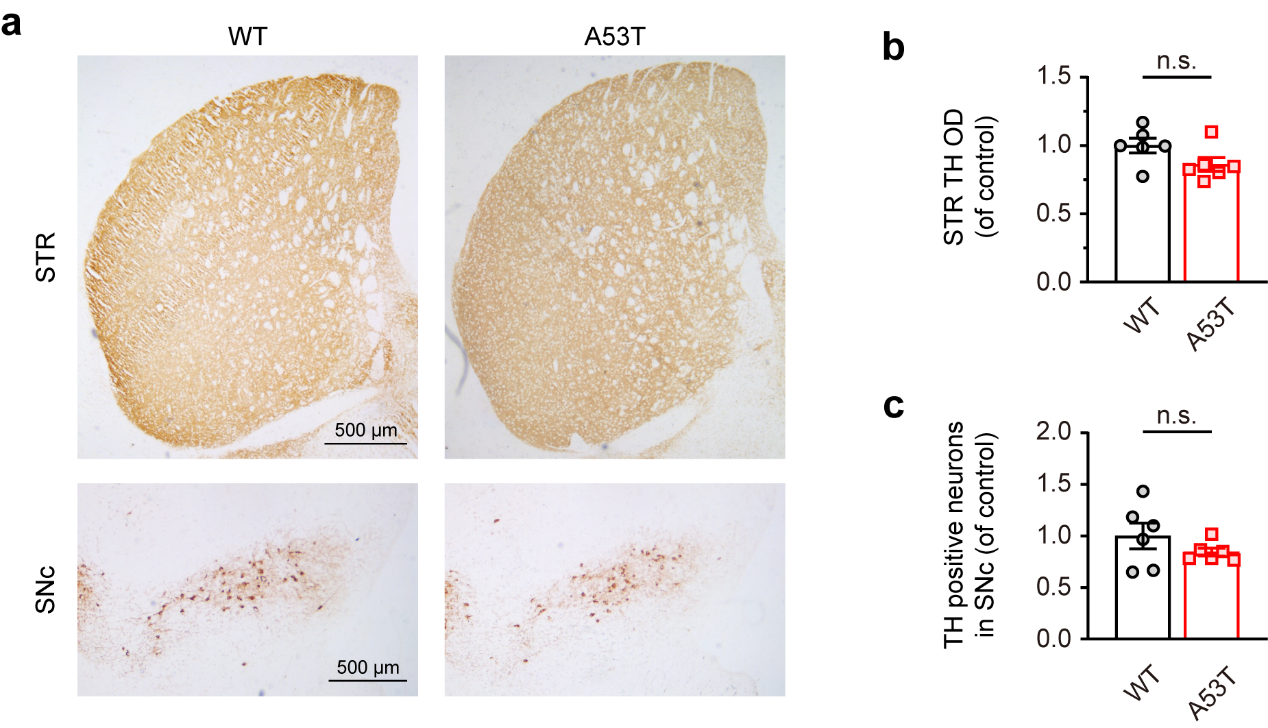
**Fig. S2** Unchanged TH-positive neurons in the STR and SNc of 6-month-old SNCA-A53T mice. **a** Representative images showing TH immunostaining of STR and SNc. **b** Quantitative analysis of the TH OD values (of control) of STR in WT and A53T mice (n=3 for each group). **c** Quantitative analysis of the number of TH-positive neurons in the SNc in WT and A53T mice (n=3 for each group). Data are presented as mean ± SEM. n.s., not significant.


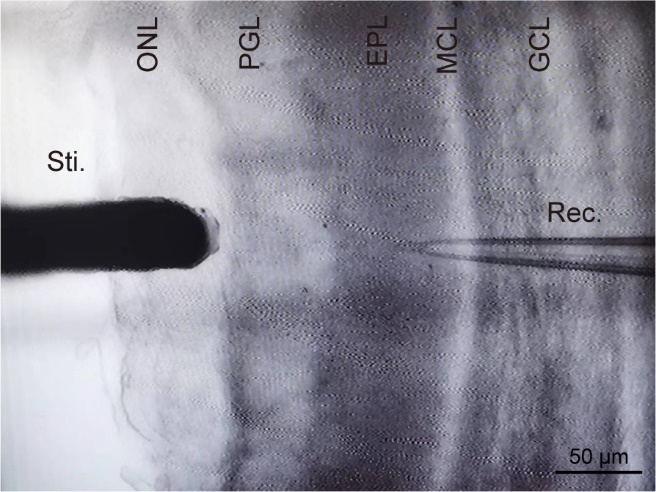


**Fig. S3** Representative image showing fEPSP recording in OB slices. ONL containing axonal terminals of OSNs was stimulated, and fEPSP was recorded in the EPL containing dendritic terminals of M/Ts in OB slices.
